# “Mega” Cisterna Chyli: A Case Report and Review of the Literature

**DOI:** 10.7759/cureus.34111

**Published:** 2023-01-23

**Authors:** Raghav Chandra, Thomas Murphy, Kirk G Jordan, John K Waters, Scott I Reznik

**Affiliations:** 1 Surgery, University of Texas Southwestern Medical Center, Dallas, USA; 2 Radiology, University of Texas Southwestern Medical Center, Dallas, USA; 3 Cardiovascular and Thoracic Surgery, University of Texas Southwestern Medical Center, Dallas, USA

**Keywords:** computerized tomography, magnetic resonance imaging, lymphangiography, thoracic duct, cisterna chyli

## Abstract

Enlarged cisterna chyli is an infrequently encountered entity and is most often an asymptomatic, incidental finding on imaging for other reasons. The pathogenesis of cisterna chyli enlargement is not well elucidated and includes infectious, inflammatory, and idiopathic causes. In this report, we present the rare case of an asymptomatic, markedly dilated “mega” cisterna chyli in a 60-year-old female.

## Introduction

Enlargement of the cisterna chyli (CC) is a rare condition and is most frequently encountered as an incidental finding on cross-sectional imaging performed for other reasons. The CC is a saccular dilatation of a lymphatic duct that receives lymphatic flow from lumbar and intestinal lymphatic trunks and serves as the origin of the thoracic duct [1]. The etiology of CC enlargement is poorly elucidated and may be a manifestation of idiopathic dilatation, primary lymphatic disease, or a result of underlying inflammatory, infectious, or traumatic pathologies [2-4]. We present an uncommon case of a benign, profoundly enlarged (12.8 x 3.4 x 2.7 cm) cisterna chyli in a 60-year-old female.

## Case presentation

**Figure 1:**
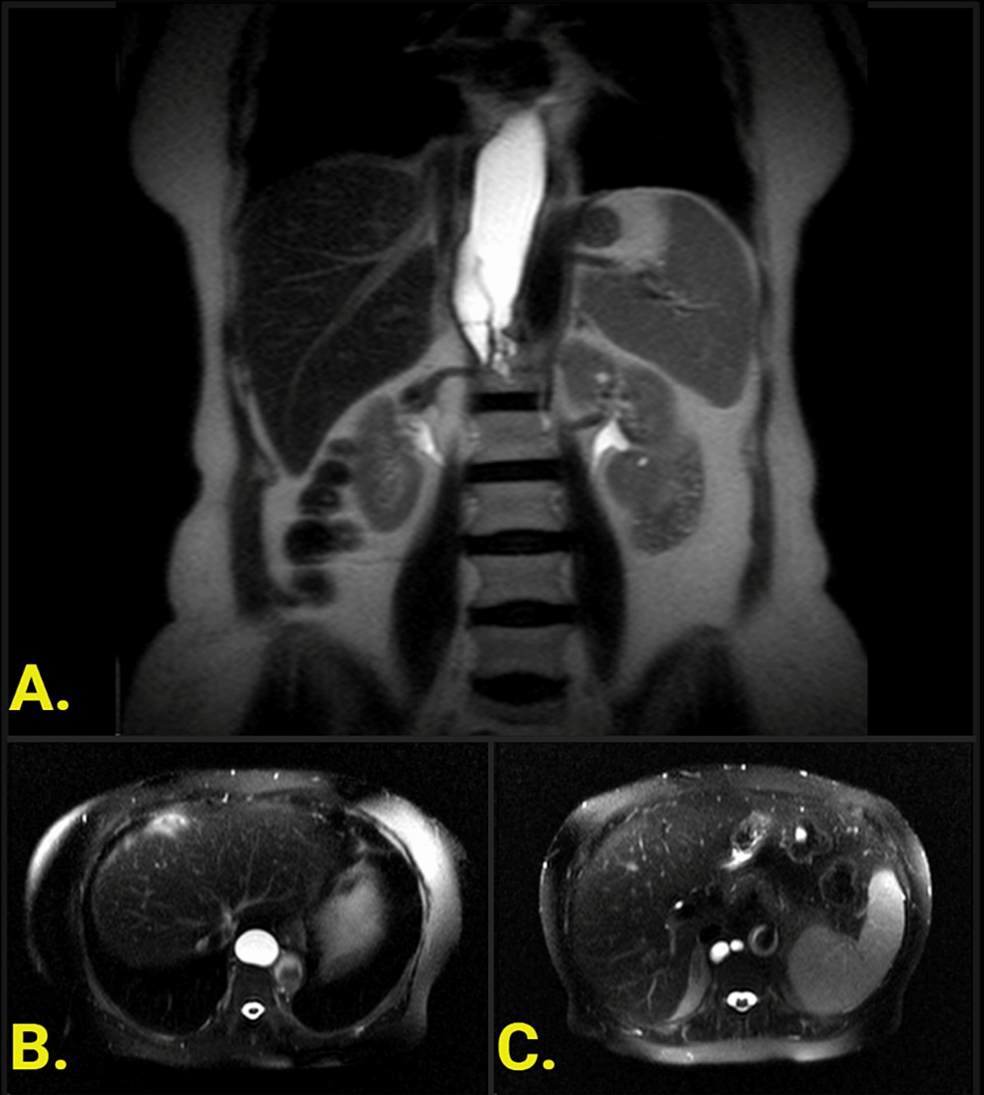
Abdominal MRI (A) Coronal and (B) Axial T2 HASTE (Half-Fourier single-shot turbo spin-echo) demonstrating uniformly hyperintense T2 signal from the enlarged cisterna chyli. (C) Coronal reformat imaging illustrates the branching feeding lymphatic channels. The lesion measured 12.8 cm craniocaudally, 3.4 cm transversely, and 2.7 cm anteroposteriorly.

**Figure 2:**
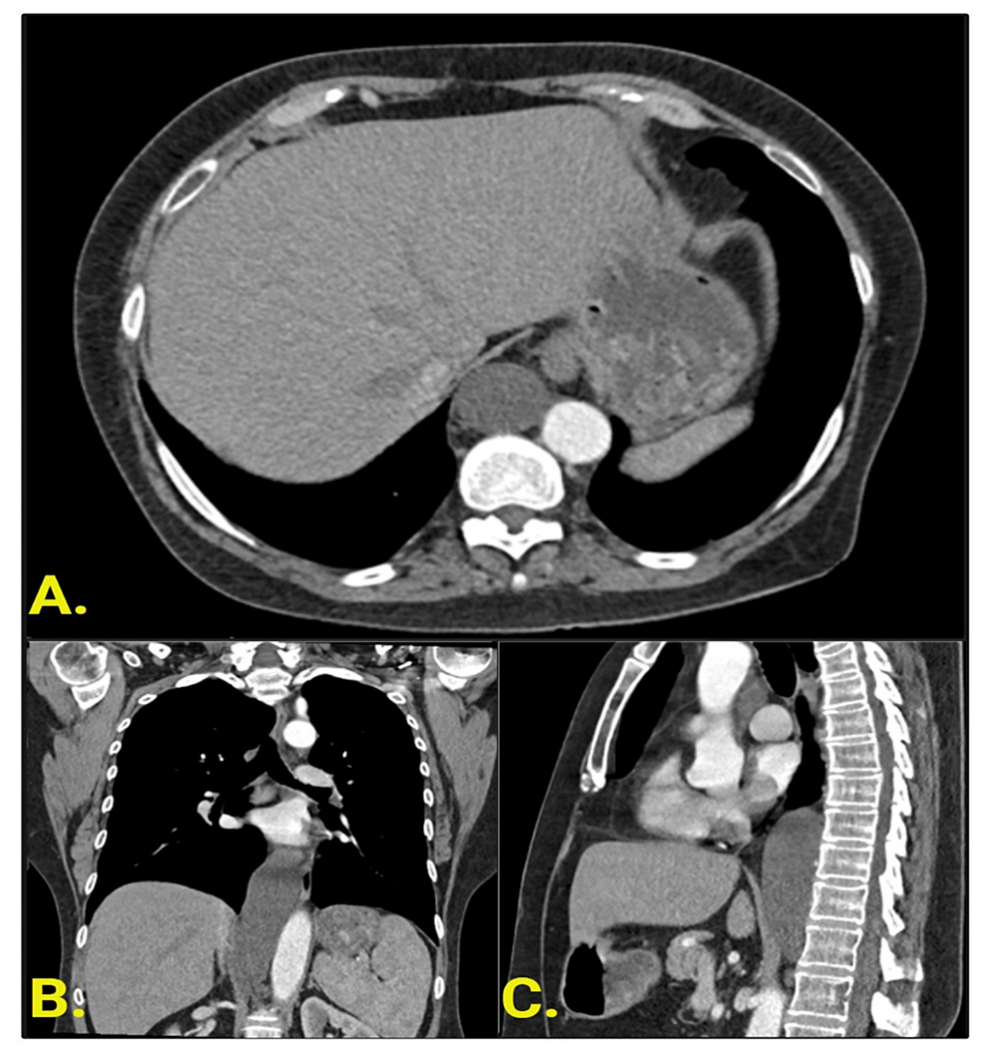
CT Chest with IV Contrast (A) Axial section demonstrating a round, well-marginated, right retrocrural lesion with homogenous attenuation (mean 13.3 HU) without enhancement and no apparent invasion of adjacent structures. (B) Coronal and (C) sagittal reformats of the same lesion demonstrate its tubular structure extending from the level of the T12 vertebral level inferiorly to the T8 vertebral level superiorly.

The patient is a 60-year-old Caucasian female with a history of hypertension, hyperlipidemia, and medically-managed Crohn’s disease who self-referred to our institution for evaluation of an incidentally encountered 12.8 cm esophageal duplication cyst. This was first discovered on a routine abdominal computerized tomography (CT) scan five years prior to her presentation. She had undergone two prior endoscopic ultrasound (EUS)-guided fine-needle aspirations (FNA) of this lesion. During the initial endoscopy, a cystic lesion under the diaphragm was identified, measuring 6 x 3 cm, thought to be a duplication cyst. EUS-FNA yielded clear, watery fluid with fluid analysis and triglyceride levels of 67 mg/dL. The cyst recurred and she underwent repeat EUS-FNA, and fluid analysis at that time demonstrated an elevated triglyceride level, 173 mg/dL. The patient denied any current symptoms including dysphagia, weight loss, or chest pain. There were no abnormal findings documented on physical examination, including no evidence of abdominal distension, ascites, or lymphedema. A presumptive diagnosis of recurrent enlarged CC was made, and the patient underwent abdominal magnetic resonance imaging (MRI) which demonstrated a large tubular cystic structure extending from the inferior mediastinum between the aorta and inferior vena cava terminating behind the crus of the diaphragm at the level of the T12/L1 vertebrae. This lesion measured 12.8 cm craniocaudally, 3.4 cm transversely, and 2.7 cm anteroposteriorly (Figure 1). The mass appeared to have thin walls and was without contrast enhancement, solid components, or diffusion restriction. These were suggestive of a benign enlarged “mega” cisterna chyli. Given these benign findings and the absence of symptoms, the plan was made to continue to observe the lesion. She underwent a repeat chest CT scan which demonstrated no significant change in the character of this cystic lesion (Figure 2).

The patient’s case was discussed in a multidisciplinary board and the decision was made to not repeat EUS-guided drainage and fluid analysis and to continue to observe the patient with serial imaging. She continues to be asymptomatic and compliant with routine screening.

## Discussion

Here, we report the highly unusual case of a markedly enlarged recurrent cisterna chyli (CC) in a 60-year-old patient. We utilized an indexed Pubmed search for keywords pertaining to the anatomy and physiology of the CC, the pathophysiology of enlarged CC, and treatment options for symptomatic disease. As no private health information was documented for this case report, informed consent was waived. 

The CC is classically described as a saccular dilatation found at the origin of the thoracic duct (TD) in the retrocrural space, most frequently found at the T11-L2 vertebral levels [1]. It serves as a reservoir for lymphatic fluid draining from the intestinal and two lumbar lymphatic trunks. A defined CC can be identified in 20% of autopsies and 50% of lymphangiographic studies [1]. Frequently, identification of the CC is difficult as its morphology is highly variable and can adopt a multifocal cystic appearance or may appear as a plexus of interconnected lymphatic channels [1]. While most CCs are found to the right of the aorta, rare cases of left-sided CC have been documented [5, 6].

Enlargement of the CC is a rarely documented occurrence, despite its heterogeneous morphology. An investigation of the CC in 40 patients through multidetector row CT (MDCT) imaging demonstrated maximal mean maximum dimensions of 3.8 mm AP x 4.0 mm TD x 14.1 mm in length (ranging from 1.7 x 7.8 x 1.3 mm to 6.9 x 3.8 x 30.1 mm) [7]. Tamsel et al. reported a case report of a CC measuring 2.5 cm AP x 3.2 cm TD x 4.2 cm in length [8]. Another case series of three patients with enlarged CC demonstrated a maximum dimension of 7 cm [9]. Furthermore, a prior study of 125 patients evaluated by MRI reported a maximum craniocaudal length of 10 cm [10]. To our knowledge, we report the largest isolated saccular CC lesion with a maximal craniocaudal length of 12.8 cm.

The etiology of enlarged CC is poorly understood and is often encountered incidentally in the diagnostic workup for other pathologies. Impaired lymphatic flow, intrathoracic lymphatic obstruction, prior radiation/trauma/injury to the thoracic duct, or increased lymph production above the capacity of the CC may be implicated. We suggest the following framework for understanding the pathogenesis of enlarged CC: (1) intrinsic: idiopathic or known weakness in lymphatic duct wall integrity or (2) extrinsic: dilatation due to increased lymph production or impaired outflow due to distal obstruction or injury of the thoracic duct or increased central venous pressure. In our case, there was no clear evidence that the patient had any intrinsic lymphangiopathy, thoracic duct pathology resulting in distal obstruction, or increased production of abdominal or lumbar lymph fluid.

Intrinsic causes of enlarged CC may be both a reflection of physiologic variations or primary lymphangiopathy. During embryogenesis, major lymphatic sacs are segmented by connective tissue to form lymph node stations, apart from the superior portion of the CC [9]. Anatomic variation in the degree and orientation of segmentation may result in variably enlarged CC. Primary lymphatic disorders including systemic lymphangiomatosis and large abdominal lymphatic malformations (LM) may also result in enlarged CC. These lesions can be multifocal [2]. Central conducting lymphatic anomalies may also be associated with impaired flow and distension through central lymphatics [11]. Infectious etiologies including filariasis can also result in secondary lymphangiectasia and subsequent CC dilation [4]. Iatrogenic injury with chylocele formation after endovascular surgery has also been reported [4].

Extrinsic causes of enlarged CC include increased lymph production beyond the capacity of the sac resulting in dilatation. Theoretically, any increased lymphatic production in this regard could precipitate enlargement of the CC. A documented example of this is cirrhosis. Verma et al. documented that cirrhosis was associated with CC dilation greater than 2 mm and this dilation was significantly more frequently encountered in uncompensated cirrhotic patients compared to those with compensated cirrhosis (54 vs. 5%, respectively) [3]. A putative mechanism for this was thought to be due to the increased production of lymph secondary to increased hydrostatic pressure and subsequent capillary filtration pressure, which may result in the distension of thin-walled lymphatic channels [12]. Conversely, malignancy does not appear to be associated with CC size; Feuerlein et al. demonstrated in a retrospective study of 711 lesions on CT imaging that CC size was not associated with cancer progression or regression [13]. There is evidence to suggest that inflammatory bowel disease (IBD), a significant chronic comorbidity in our patient’s case, may be associated with lymphatic abnormalities including intralymphatic lymphocytic thrombi, granulomatous lymphangitis and poor lymphatic drainage [14]. Furthermore, Geleff et al. reported that the distribution of podoplanin-positive lymphatic microvessels in resected small bowel specimens was significantly enriched in the muscularis mucosa, submucosa, and subserosa compared to uninvolved areas of the intestine, a finding corroborated by Rahier et al. who demonstrated significantly increased lymphatic density in patients with Crohn’s disease compared to control patients [15,16]. It is plausible that these may reflect increased lymph production, possibly in response to chronic inflammation. Thus, comorbid IBD could potentially be a cause of CC dilatation.

Symptomatically enlarged CC are rare, and most lesions are found incidentally on imaging for other indications. Rarely, enlarged CC can result in pain, gastrointestinal discomfort, nausea, or weight loss [8, 17]. In younger patients with large abdominal LMs, these may be associated with life-threatening complications including dysmotility, distal obstruction, chylothorax/ascites, and protein-losing enteropathies [2]. In the present case, the patient reported no associated symptoms including chest or abdominal pain, weight loss, or dysphagia.

The diagnosis of enlarged CC should be made at the exclusion of other pathologies. Key imaging characteristics may aid in narrowing the differential diagnoses. These include retrocrural lymphadenopathy in the setting of malignancy or infection [4,8]. The imaging differential diagnosis for cystic mediastinal and retrocrural masses is broad and includes esophageal duplication cysts, benign congenital cysts, meningocele, cystic schwannoma, mature cystic teratoma, lymphangioma, abscess, pancreatic pseudocysts, cystic degeneration of malignant tumors, as well as normal anatomic variants [18-20]. Detailed anatomic location on cross-sectional imaging, tissue characteristics, as well as enhancement pattern are all critical in distinguishing these entities. For this reason, the imaging workup generally includes contrast-enhanced CT and often subsequent contrast-enhanced MRI. Imaging features that suggest a benign cystic etiology have been well-described and include smooth contour with well-defined margins, homogenous attenuation (0-20 Hounsfield Units), no enhancement of internal cystic contents, no infiltration of adjacent structures, as well as low T1 and high T2 signal [19].

The normal appearance of the CC on routine MRI of the abdomen has also been well-described [1]. In this series, the CC was reliably seen in 15% (30/200) of patients undergoing routine abdominal MRI. The authors describe the normal CC as being a right retrocrural lesion, variably shaped with the most common being a branched inverted V or Y shape, fluid-filled, non-enhancing, and centered at the T12-L2 vertebral levels extending 5-7 cm in a caudocephalic direction [1].

There are multiple features in this case that suggested a benign etiology; the lesion was rounded with a smooth contour and well-defined margins, with no evidence of invasion of adjacent mediastinal or retrocrural structures, exhibited no internal enhancement, and was of homogenous fluid attenuation (Figure 1). This lesion can be further delineated with a high likelihood as an enlarged CC given its appearance on MRI (Figure 1). The defining features include a uniformly hyperintense T2 signal, uniformly hypointense T1 signal, and absent enhancement pattern. A particular feature of the lesion consistent with enlarged CC was the branched appearance of numerous lumbar lymphatics draining into the saccular lesion, as well as its inverted Y-shape.

As the vast majority of enlarged CCs are asymptomatic, treatment is seldom required and infrequently reported in the literature. Rare complications including persistent chylothorax, chylous ascites, or gastrointestinal symptoms may necessitate intervention. Conservative approaches include chest tube drainage, medium-chain fatty acid diet, parenteral nutrition, and somatostatin analogue therapy (octreotide) [21]. No reports of interventional radiographic procedures specifically for enlarged CC were identified. Thoracic duct embolization (TDE) is a well-described technique for postoperative and nontraumatic chylothoraces with highly variable success rates ranging from 33-100% [22]. This technique typically involves transpedal or intranodal lymphangiography to first identify the lymphatic system and select a target vessel for catheterization. It is recommended to target a lumbar lymphatic vessel below the CC as an access point, given the risk of a chyle leak if the CC is directly accessed. The chyle leak is then identified fluoroscopically and the thoracic duct is embolized proximal to the identified leak. Ultimately in this case, embolization of the thoracic duct causing lymphatic obstruction would likely lead to further dilation of the CC, and therefore, provide little benefit.

While transthoracic surgical ligation of the TD is well-established, surgical ligation of the CC is rarely performed and reserved for chylothorax or ascites refractory to conservative or radiographic management, often in pediatric cases [23, 24]. Zanin et al. described a case of chronic bilateral chylothoraces after congenital heart surgery in a 22-month-old female. After repeated hospitalizations, the patient underwent an open ligation of the CC through an upper midline celiotomy with a marked reduction in chyle leak and rapid symptomatic improvement [25]. Diaz-Gutierrez et al. [23] recently reviewed their minimally invasive experience with three cases of refractory chylothorax in patients with idiopathic or postoperative chylothorax refractory to TDE, surgical ligation, and with contraindications to radiographic intervention. The authors described their laparoscopic approach to CC ligation as consisting of a five-port approach, exposure of the right lateral aorta, dissection of the right crus to the inferior vena cava, exposure of the CC posteriorly, and ligation. Postoperatively, two out of three patients exhibited a significant reduction in chest tube output [23]. To our knowledge, radiographic ablation or surgical ligation of an asymptomatic enlarged CC has not been performed and we suggest that even lesions as large as the one reported in this case can be observed expectantly in the absence of symptoms.

In addition to upfront ligation, Ishiura et al. [2] reported their experience managing a large abdominal LM complicated by chylous ascites protein-losing enteropathy, and lymphedema in a 10-year-old female with lymphovenous anastomosis. Preoperative lymphoscintigraphy demonstrated multifocal LMs with obstruction at the CC. Intraoperatively, after the evacuation of more than 12 liters of ascitic fluid, three microsurgical lymphovenous bypasses were performed between LMs and bilateral ovarian veins, as well as between an LM branch around the CC to an omental vein.

## Conclusions

We report here the rare case of a 60-year-old female incidentally found to have an extremely enlarged (12.8 x 3.4 x 2.7 cm) "mega" cisterna chyli, manifesting as a recurrence after prior endoscopic drainage. Enlargement of the cisterna chyli is extremely uncommon. While most cases are idiopathic, intrinsic and extrinsic factors may predispose CC dilatation. Most enlarged CCs can be monitored. In rare symptomatic cases of enlargement (commonly associated with pediatric lymphatic malformations) or in the setting of iatrogenic injury or pathologic obstruction, conservative management consists of conversion to medium chain fatty-acid diet, parenteral nutrition, chest tube drainage, and somatostatin analogue therapy. Refractory cases may be amenable to radiographic embolization. Rarely, surgical ligation of the CC, through open or laparoscopic approaches, or lymphovenous bypass may be necessary.
